# Exploring Caregivers’ Unmet Needs, Unmet Needs Distress, and Well-being: The Role of Personal Gain and Mastery

**DOI:** 10.1093/geroni/igaf122.568

**Published:** 2025-12-31

**Authors:** Emma Jackson, Claire Grant, Lauren Stratton, Katherine Judge

**Affiliations:** Cleveland State University, Westlake, Ohio, United States; VA, Waltham, Massachusetts, United States; Alzheimer’s Association, Chicago, Illinois, United States; Cleveland State University, Cleveland, Ohio, United States

## Abstract

Informal dementia caregivers experience a range of negative well-being outcomes and reported unmet needs and related distress. Few studies have examined how caregiver’s unmet needs and corresponding distress may contribute to well-being along with intrapersonal factors as mediators. Guided by the Stress Process Model, this paper examined the mediating role of personal gain and mastery (intrapersonal factors) in the relationship between well-being outcomes (depression and anxiety), unmet needs, and unmet needs distress using 4 parallel mediation models. Of the 440 participants (Mage = 48.94), 68.9% were female and 58.6% White. Neither personal gain nor mastery were found to be significant mediators for unmet needs and the well-being outcomes. For unmet needs distress predicting depression, the indirect effect for personal gain was significant (b = -.0290, CI [-.0633, -.0022]) while mastery was not (b = -.0549, CI [-.1233, .0033]). Similarly, for unmet needs distress predicting anxiety, personal gain was significant (b = -.0364, CI [-.0721, -.0102]) while mastery was not (b = -.0503, CI [-.1112, .0017]). Results indicated 1) intrapersonal factors did not mediate the relationship between unmet needs and well-being outcomes and 2) personal gain mediated the relationship between unmet needs distress and both well-being outcomes. These findings suggest the constructs of unmet needs and unmet needs distress function differently and for unmet needs distress, personal gain rather than mastery, was important in understanding the relationship with depression and anxiety. These findings are important for expanding our theoretical understanding of the stress process along with implications for interventions.

